# Sebaceous gland abnormalities in fatty acyl CoA reductase 2 (*Far2*) null mice result in primary cicatricial alopecia

**DOI:** 10.1371/journal.pone.0205775

**Published:** 2018-10-29

**Authors:** John P. Sundberg, Tong Shen, Oliver Fiehn, Robert H. Rice, Kathleen A. Silva, Victoria E. Kennedy, Nicholas E. Gott, Louise A. Dionne, Lesley S. Bechtold, Stephen A. Murray, Raoul Kuiper, C. Herbert Pratt

**Affiliations:** 1 The Jackson Laboratory, Bar Harbor, Maine, United States of America; 2 West Coast Metabolomics Center, University of California, Davis, California, United States of America; 3 Biochemistry Department, King Abdulaziz University, Jeddah, Saudi-Arabia; 4 Department of Environmental Toxicology, University of California, Davis, California, United States of America; 5 Department of Laboratory Medicine, The Karolinska Institute, Stockholm, Sweden; University of Florida, UNITED STATES

## Abstract

In a large scale screen for skin, hair, and nail abnormalities in null mice generated by The Jackson Laboratory’s KOMP center, homozygous mutant *Far2*^*tm2b(KOMP)Wtsi*^/2J (hereafter referrred to as *Far2*^*-/-*^) mice were found to develop focal areas of alopecia as they aged. As sebocytes matured in wildtype C57BL/NJ mice they became pale with fine, uniformly sized clear lipid containing vacuoles that were released when sebocytes disintegrated in the duct. By contrast, the *Far2*^*-/-*^ null mice had sebocytes that were similar within the gland but become brightly eosinophilic when the cells entered the sebaceous gland duct. As sebocytes disintegrated, their contents did not readily dissipate. Scattered throughout the dermis, and often at the dermal hypodermal fat junction, were dystrophic hair follicles or ruptured follicles with a foreign body granulomatous reaction surrounding free hair shafts (trichogranuloma). The Meibomian and clitoral glands (modified sebaceous glands) of *Far2*^*-/-*^ mice showed ducts dilated to various degrees that were associated with mild changes in the sebocytes as seen in the truncal skin. Skin surface lipidomic analysis revealed a lower level of wax esters, cholesterol esters, ceramides, and diacylglycerols compared to wildtype control mice. Similar changes were described in a number of other mouse mutations that affected the sebaceous glands resulting in primary cicatricial alopecia.

## Introduction

Primary cicatricial (scarring) alopecia (PCA) is a term that encompasses a group of human diseases historically believed to be due to an inflammatory or autoinflammatory skin disease process leading to follicle destruction, fibrosis, and loss of stem cells in the bulge region, ultimately resulting in follicular scars. These can be primary or secondary; the primary forms often are associated with damage to sebaceous glands [1]. A number of mutant mouse strains provide genetic evidence to support a unifying role for altered sebum homeostasis in this pathogenesis. This was first described in the asebia mouse strain (stearoyl-coenzyme A desaturase 1, *Scd1*) and shortly thereafter in what were later shown to be an allelelic series in bareskin, defolliculated, and finnigan (gasdermin A3, *Gsdma3*) [2–4] mutant mice, with more attention recently on a variety of conditional null alleles of peroxisome proliferator activated receptor gamma (*Pparg*) [5, 6]. It was therefore not surprising to find that these and many other lipid metabolizing enzymes interact directly or indirectly with each other in a complex molecular pathway [1]. As for many diseases with a genetic basis, other genes in these pathways, when mutated, can result in similar lesions. Together these mouse models provide useful clues to the complex pathogenesis of some forms of human primary cicatricial alopecias.

In a survey looking for skin abnormalities in genetically engineered mice, the Knockout Mouse Project (KOMP), mice with patchy alopecia were identified that lacked function of the fatty acyl coA reductase 2 (*Far2*) gene [7]. An abnormal hair phenotype for these mutant mice was reported in the mouse phenotype database for the KOMP project but no details were provided on the skin or hair abnormalities associated with the alopecia other than graphs on lesion distribution (http://www.mousephenotype.org/data/search/gene?kw=%22Far2%22; accessed 8 Apr 2018).

We describe here abnormalities of the sebaceous glands and skin surface lipids associated with follicular dystrophy leading to follicular scarring without direct effects on the hair shafts in these mutant mice.

## Materials and methods

### Mice

**Fig 1:**
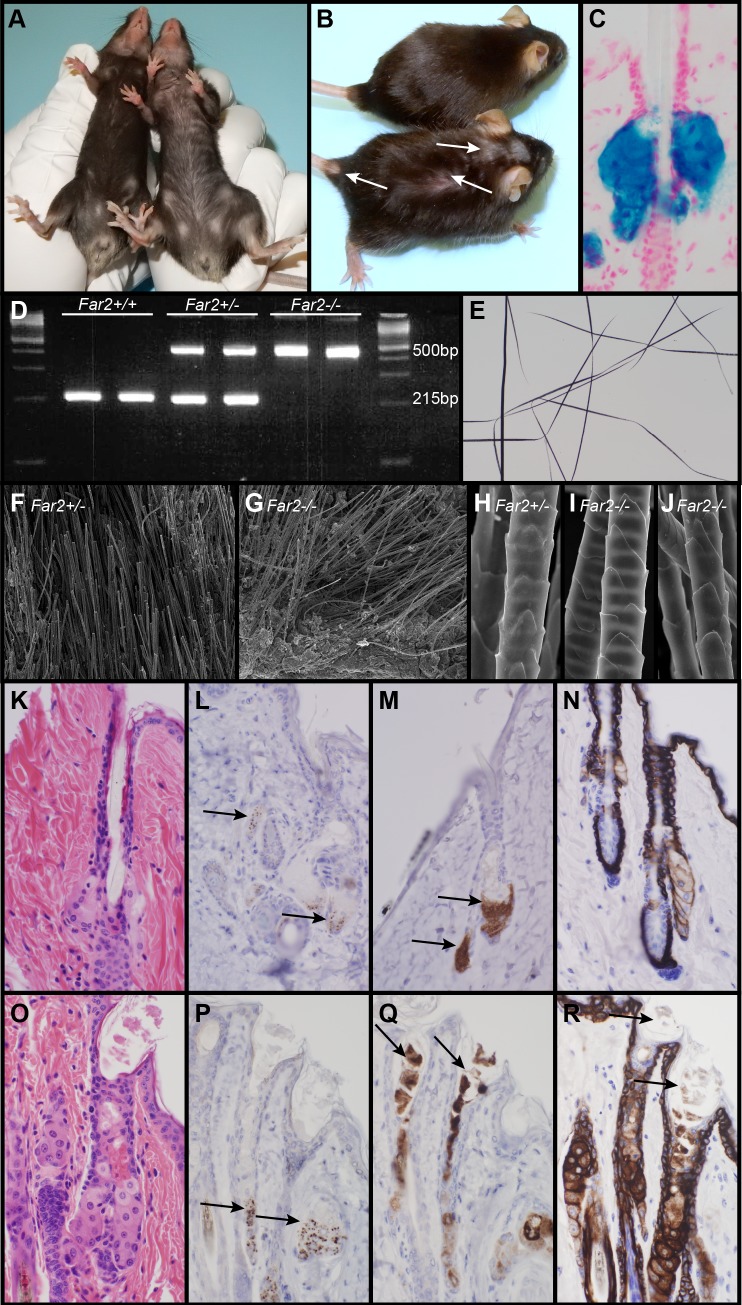
Features of *Far2*^-/-^ mice. Heterozygous mouse (A, left; B, top) with a normal thick black hair coat. By contrast, a homozygous *Far2*^*-/-*^ mouse (A, right; B, bottom) had focal alopecia on the top of its head behind the ears. *Far2*^*tm1(KOMP)Wtsi*^/2J mice (normal with a LacZ reporter gene) expressed LacZ only in sebaceous glands (C). Genotyping differentiates wildtype (*Far2* ^+/+^), heterozygous (*Far2*^+/-^), and mutant (*Far2*^-/-^) mice (D). Plucked hair shafts taken from a *Far2*^-/-^ mouse adjacent to areas of alopecia were indistinguishable from normal (E). By scanning electron microscopy there were no obvious structural differences in hair shafts between the normal heterozygous (F) or null (G, I, J) *Far2* mice in either whole skin mounts with hair (F, G) or plucked hairs mounted in groups. Histologically, wildtype sebaceous glands had pale cytoplasm with fine clear vacuoles, the contents of which could not be seen in routine sections once the cells ruptured (K). By contrast, sebaceous glands in *Far2*^*-/-*^ mice had bright eosinophilic cytoplasm that, when ruptured into the sebaceous duct, formed clumps of amphophilic material (O). SOAT1 immunolabeled sebocytes at the base of the sebaceous gland in both genotypes (L, P). Perilipin 2 (PLIN2; also called adipophilin) labeled the sebocytes near the base of the gland in normal mice (M) but also labeled the extruded material within the infundibulum and sebocytes in the *Far2* null mice (Q). Keratin 14 (KRT14) normally labels basal cells and hair follicle root sheath cells with weak cell membrane labeling of sebocytes (N), but in the *Far2*^*-/-*^ mice sebocytes were heavily labeled as was the extruded material within the infundibulum (R).

Fatty acyl coA reductase 2 (*Far2*^*-/-*^) null (B6N(Cg)-*Far2*^*tm2b*^*(KOMP)Wtsi*/2J), C57BL/6NJ (wildtype control), and *Far2*^*tm1(KOMP)Wtsi*^/2J mice (histologically normal with a LacZ reporter gene) mice were obtained from The Jackson Laboratory, Bar Harbor, ME. Mice were genotyped by PCR using defined protocols (Fig 1D) (https://www2.jax.org/protocolsdb/f?p=116:5:0::NO:5:P5_MASTER_PROTOCOL_ID,P5_JRS_CODE:24558,022011; accessed 10 Apr 2018).

Mice were maintained in the humidity, temperature, and light cycle (12:12) controlled vivarium under specific pathogen-free conditions (http://jaxmice.jax.org/genetichealth/health_program.html) and were housed in double-pen polycarbonate cages (330 cm^2^ floor area) at a maximum capacity of four mice per pen. Mice were allowed free access to autoclaved food (NIH 31, 6% fat; LabDiet 5K52, Purina Mills, St. Louis, MO) and acidified water (pH 2.8–3.2). All work was done with the approval of The Jackson Laboratory Animal Care and Use Committee under approval numbers 07005 and 99066.

### Histopathologic analyses

Mice were euthanized by CO_2_ asphyxiation followed by open chest necropsy. Twelve mice were necropsied, 8 females and 4 males 172 to 644 days of age. Dorsal and ventral truncal skin, ear skin, tail skin, eyelids, muzzle, and digits (to include the foot pads and nail unit) were fixed by immersion in Fekete’s acid-alcohol-formalin for 12 hours, trimmed, processed routinely, sectioned at 5 μm, and stained with hematoxylin and eosin (H&E) [8]. Clitoral glands were examined in two 15 week old females. In addition, hair was plucked and stored in screw topped tubes and 1 cm^2^ of dorsal haired skin (adjacent to the area of alopecia) was removed and fixed in buffered glutaraldehyde for scanning electron microscopy (see below) from one homozygous and one heterozygous 287 day old female mouse.

### *Far2* normal gene expression

*Far2*^*tm1(KOMP)Wtsi*^/2J mice (histologically normal with a LacZ reporter gene) were used to determine the tissue and/or cell type specific distribution of *Far2* gene expression in adult, juvenile, and fetal mice using methods previously described [9, 10]. Additional images are available on the Mouse Genome Informatics website (http://www.informatics.jax.org/marker/key/94785; accessed 10 Apr 2018).

### Immunohistochemistry

Immunohistochemistry was performed on serial sections of dorsal skin from 2 female and 2 male *Far2*^-/-^ mice 26 weeks of age and one female and one male control of the same age using a Ventana Discovery XT autostainer (Ventana, Tuscon, AZ) for cleaved caspase 3 (CASP3, 1:800, stock #9661, Cell Signaling Technology, Danvers, MA), sterol O-acyltransferase 1 (SOAT1, 1:200, stock#ab39327, Abcam, Cambridge, MA), Perilipin 2 (PLIN2, also called apidophilin, 1:200, cat#GP40, Progen, Heidelberg, Germany), and Keratin 14 (KRT14, 1:10,000, stock#PRB-155P, Covance, Berkeley, CA), as described previously [11, 12]. Diaminobenzidine (Sigma, St. Louis, MO) was used as chromogen for all antibodies except for Perilipin 2, which used with the avidin-biotin method and the Bluemap kit (cat#760–120, Ventana).

### Electron microscopy

Skin was collected from the dorsal cervical region adjacent to the sites of alopecia from one female B6N(Cg)-*+/Far2*^*tm2b(KOMP)Wtsi*^/2J (heterozygous grossly normal mouse) and one female B6N(Cg)-*Far2*^*tm2b(KOMP)Wtsi*^*/Far2*^*tm2b(KOMP)Wtsi*^/2J (homozygous mutant mouse) that were 287 days of age at the time of necropsy. Standard methods are described in detail elsewhere [13–15].

For scanning electron microscopy (SEM), a 2.0 cm^2^ sample of dorsal skin adjacent to the area of alopecia was removed at the time of necropsy, placed with the hair side up on nylon mesh, immersed in cold 2.5% glutaraldehyde in 0.1M cacodylate buffer, and fixed overnight at 4^o^ C. Samples were then washed 2X in 0.1M cacodylate buffer and post fixed in 0.5% osmium tetroxide in 0.1M cacodylate buffer. Samples were dehydrated through a series of graded ethanols to 100%, after 3 changes in 100% ethanol, samples were dried using hexamethyldisilazane (HMDS), attached to aluminum stubs with silver adhesive, sputter coated with 15 nm of gold, and examined in an Hitachi S3000N scanning electron microscope (Hitachi Corp., Schaumburg, IL) operated at 20 kV. Plucked hairs were attached to aluminum stubs and processed in the same manner.

For transmission electron microscopy (TEM), skin from the affected area is finely sliced into strips with razor blades, fixed by immersion for 18 hours in 2.5% glutaraldehyde in 0.1M cacodylate buffer (pH 7.4), and post fixed for 18 hours in aqueous 1% osmium tetroxide. Tissues were stained *en bloc* in 2% uranyl acetate in 10% ethanol then dehydrated through graded ethanols. Skin was embedded in Spurrs-Mollenhauer resin and polymerized at 65°C for 48 hours; ultrathin sections were collected, stained with uranyl acetate and lead citrate, and examined in a JEOL 1230 transmission electron microscope (JEOL Corp., Tokyo, Japan) operated at 80kV.

### Lipidomic analyses

Sebum was collected from 5 females and 5 males ranging in age from 8 to 24 weeks from C57BL/6NJ (*Far2*^+/+^, wildtype) and B6N(Cg)-*Far2*^*tm2b(KOMP)Wtsi*^/2J (*Far2*^-/-^) mouse strains. Briefly, mice were euthanized, then dorsal skin between the shoulder blades of the mice was shaved with electric clippers. The skin was stretched in a metal round tart mold with a 2 cm diameter hole in the bottom. A euthanized mouse was placed in a mold to make a tight seal and to expose the shaved, dorsal lumbar skin from the hole. The exposed skin was then immersed in 5 mL of acetone in a glass petri dish and swirled for 20 seconds. The acetone was then transferred into a glass tube and a second wash of acetone was used to wash the petri dish and this was added to the same glass tube. Using a 9-port Reacti-Vap Evaporator (VWR, Radnor, PA), a low flow of nitrogen (≥7 p.s.i) was sent through the tubes to dry the acetone leaving only the lipids at the base. The samples were then sealed with a rubber stopper and parafilm and stored refrigerated until used.

Sebum lipids consist of glycerides (diacylglycerols, DGs; triacylglycerols, TGs), wax esters (WE), squalene, cholesterol esters (CEs), as well as free cholesterol, and fatty acids (FAs) [16]. Lipidomics analysis was conducted using a sensitive UHPLC-MS/MS data dependent acquisition as described [17], which analyzed lipid classes of TGs, DGs, CEs, FAs, glycerolphospholipids (GPLs or PLs), (lyso)glycerolphospholipids ((lyso)PLs), ceramides (Cers), and sphingomyelins (SMs). Wax esters were analyzed separately by a modified UHPLC method [18]. Both the lipidomics and wax esters analyses were conducted on a UHPLC 1290 Infinity Binary LC system (Agilent Technologies, Santa Clara, CA, USA) with a charged surface hybrid (CSH) C18 column (Acquity CSH C18 column; 1.7mm 2.13 100 mm; Waters, Milford, MA,USA) and Agilent 6530 QToF mass spectrometer (Santa Clara, CA). For the lipidomics analysis, mobile phase A consisted of 60:40 v/v acetonitrile:water and mobile phase B consisted of 90:10 v/v isopropanol:acetonitrile. Ammonium formate (10 mM) and formic acid (0.1% v/v) were added equally to both mobile phases as positive-ion mode modifiers, and ammonium acetate (10 mM) was used for negative-ion mode analysis. Gradient program for both positive- and negative-ionization mode was as follows: 15% B (initial), gradient to 30% B at 2.0 min, gradient to 48% B at 2.5 min, gradient to 82% B at 11.0 min, gradient to 99% B at 11.5 min, hold at 99% B until 12.0 min, gradient to initial conditions at 12.1 min, and hold until 15 min. Flow rate was 0.6 ml/min. Sample (1.67μl) was injected in positive and 5 μl in negative mode. For the analysis of wax esters, mobile phase A consisted of 5mM Ammonium Formate and mobile phase B consisted of 95:5 v/v methanol:isopropanol. Gradient program for both positive- and negative-ionization mode was as follows: 70% B (initial), gradient to 99% B at 0.5 min till 12 min, gradient to 70% B at 70 min, and hold until 15 min. Flow rate was 0.6 ml/min. Samples were reconstituted with 2:1 v/v methanol:chloroform. Data acquisition was conducted with the following parameters: capillary voltage, 3500 V; drying gas flow rate, 15 L/min; gas temperature, 250°C; nozzle voltage, 1000 V; sheath gas flow rate, 11 L/min; sheath gas temperature, 350°C; full scan MS1 scan rate, 4 scans/sec; data-dependent tandem MS (MSMS) (top 4 abundant ions) scan rate, 8 scans/sec. Pools of each sample group were created and injected to collect tandem MS data for annotation purposes. Electrospray ionization setting and liquid chromatography were optimized for detecting wax esters. Metabolites were annotated in MS-DIAL (v2.78) by matching accurate mass (MS1), MS/MS, and retention time with built-in libraries. Wax esters were manually annotated [16].

## Results

*Far2*^-/-^ mice developed hair loss consistently at 4 months of age initially on the ventral skin where it presented as a gradual thinning of hair. Patchy hair loss initially was observed on the top of the head between the ears that later extended to the interscapular region of the dorsal trunk (Fig 1A and 1B).

In truncal skin from *Far2*^*tm1(KOMP)Wtsi*^/2J mice (normal mice with a LacZ reporter gene to localize *Far2* expression), LacZ expression was restricted to sebaceous glands (Fig 1C). Modified sebaceous glands were not tested. PCR analysis using standard protocols (https://www.jax.org/strain/022011) revealed a single band at 215bp for wildtype mice, bands at 215bp and 500bp for heterozygous mice, and one band at 500bp for *Far2* null mice (Fig 1D).

*Far2*^*-/-*^ mice had normal appearing hair shafts in mounts of plucked hair examined using white light (Fig 1E) and scanning electron microscopy (Fig 1H–1J). Hair shafts *in situ* on dorsal skin were also indistinguishable from heterozygous mice by scanning electron microscopy (Fig 1F and 1G).

**Fig 2:**
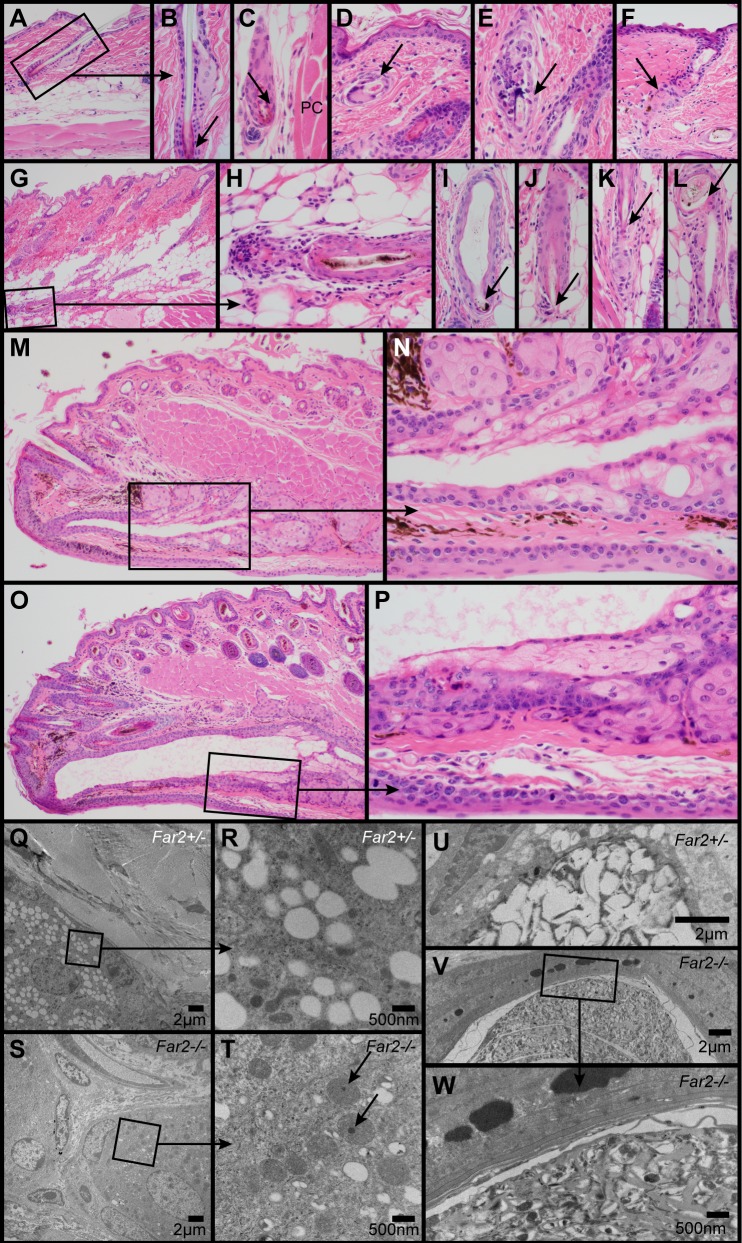
Histologic and ultrastructural changes in the skin. Normal telogen follicle in a control mouse illustrates the normal sebaceous gland and club hair (arrow, A, B). In *Far2*^*-/-*^ mice occasional telogen hair follicles (arrow) were present deep in the hypodermal fat layer adjacent to the panniculus carnosus (PC) muscle (C). Follicular dystrophy with or without rupture and associated inflammation was present in the dermis (arrow, D, E) and hypodermal fat layer (G-L). Occasional follicular scars (arrow, F) were present extending from below the sebaceous gland. Meibomian glands in the eyelids of wildtype mice (M, N) had a mildly dilated duct that was markedly dilated in *Far2*^*-/-*^ mice (O, P). Ultrastructurally, a normal sebocyte in a female, 287 day old *Far2*^*+/-*^ heterozygote mouse had numerous clear cytoplasmic vacuoles (Q). Higher magnification of boxed area (R) revealed the details of these vacuoles and surrounding organelles. By contrast, sebocytes in an age and sex matched *Far2*^*-/-*^ homozygote mutant mouse had very few clear vacuoles and prominent oval mitochondria (S), some with electron dense material (arrow; T). A normal sebocyte in the sebaceous gland duct of a normal heterozygous female had numerous clear cytoplasmic vacuoles that were being compressed and ruptured (U). By contrast, sebocytes in an age and sex matched *Far2*^-/-^ mouse had very few clear vacuoles and abundant cellular debris extending into the follicular infundibulum (V, W).

Sebaceous glands usually vary in size throughout the hair cycle in mice [19–21] but have similar morphologic features throughout life as was the case for wildtype and heterozygous mice in this study. In young *Far2*^*-/-*^ mice hair follicles appeared to be relatively normal with subtle differences. In control mice the reserve cells at the base of the sebaceous glands mature to have abundant pale cytoplasm with fine pale vacuoles. When mature sebocytes disintegrate within the sebaceous gland duct, which is normal for this holocrine gland, their remains and cytoplasmic content constitute a waxy secretum that is not visible in routine H&E stained sections (Figs 1K and 2A and 2B).[22] By contrast, *Far2*^-/-^ mice hair follicles had infundibula that were mildly to moderately dilated (ectatic). Mature sebocytes initially looked normal but as they entered the sebaceous gland duct, sebocyte cytoplasm became brightly eosinophilic compared to the light pale staining cells in controls processed concurrently in H&E stained sections. Mutant mouse sebocytes did not fully disintegrate as they entered the sebaceous gland duct and what appeared to be clustered eosinophilic remnants extended into the infundibula (Fig 1O).

Club hairs in telogen follicles are normally present in the dermis (Fig 2A). In the older *Far2*^-/-^ mice, club hair and the dermal papilla of telogen hair follicles were occasionally found deep in the hypodermal fat layer adjacent to the panniculus carnosus muscle (Fig 2C). These were found in areas where hair follicles were much longer than normal. Follicular dystrophy, where hair follicle root sheaths underwent metaplasia to the anatomic features of epidermis and contained abnormal hair shafts, were associated with penetration of the hair shaft through the follicle wall, with an associated mixed inflammatory cell infiltrate often containing numerous macrophages and multinucleated giant cells (trichogranulomas, Fig 2D, 2E, 2G and 2L). Occasional follicular scars were found either extending from below sebaceous glands in the dermis (Fig 2F) or in the hypodermal fat layer (Fig 2G, 2H and 2K).

Meibomian (Fig 2M and 2P) and clitoral glands (not shown), both specialized sebaceous glands in the mouse, were histologically similar in both *Far2*^*-/-*^ and wildtype mice but the duct leading to the surface of the mucocutaneous junction was markedly ectatic in the mutant mice.

Immunohistochemistry was undertaken to examine changes in sebaceous gland markers in the *Far2* mutant mice. Sterol O-acyltransferase 1 (SOAT1) was expressed in the reserve cells of the sebaceous glands in both mutant and wildtype mice in a similar pattern (Fig 1L and 1P). Wildtype mice expressed perilipin 2 (PLIN2, also called adipophillin) only in the reserve cells and early differentiated sebocytes (Fig 1M). By contrast, PLIN2 was expressed in seboctyes and their excreted cytoplasmic contents within the infundibulum in the *Far2* mutant mice (Fig 1Q). Keratin 14 (KRT14) labeled epidermis, root sheaths, and sebocytes in both mutant and wildtype mice; however, the excreted contents of mutant sebocytes also were labeled with this marker (Fig 1N and 1R). Cleaved caspase 3 (CASP3) labeled apoptotic keratinocytes in *Sharpin*^*cpdm/cpdm*^ mutant mice that have a hyperplastic epidermis with an abundance of apoptotic keratinocytes [23]. There were no positive cells identified in the *Far2*^*-/-*^ epidermis, hair follicles, or sebaceous glands.

Ultrastructural evaluation of the skin revealed sebocytes in a normal *Far2* heterozygote mouse with numerous clear cytoplasmic vacuoles (Fig 2Q and 2R). By contrast, sebocytes in a *Far2* null mouse had very few clear cytoplasmic vacuoles, and those that were present varied in size but overall were smaller than those in normal mice (Fig 2S and 2T). Sebocytes that entered the sebaceous gland duct or hair follicle infundibulum normally disintegrate and extrude their cytoplasmic contents which appeared ultrastructurally as clear cytoplasmic vacuoles that became irregular and coalesced as the sebocytes matured (Fig 2U). By contrast, sebocytes in a *Far2*^*-/-*^ mouse had very few clear vacuoles. Their cytoplasmic contents contained irregular material in size, shape, and electron density (Fig 2V and 2W). Mitochondria appeared swollen and occasionally contained occasional small, solitary, electron dense granules which were not found in the control (Fig 2T).

**Fig 3:**
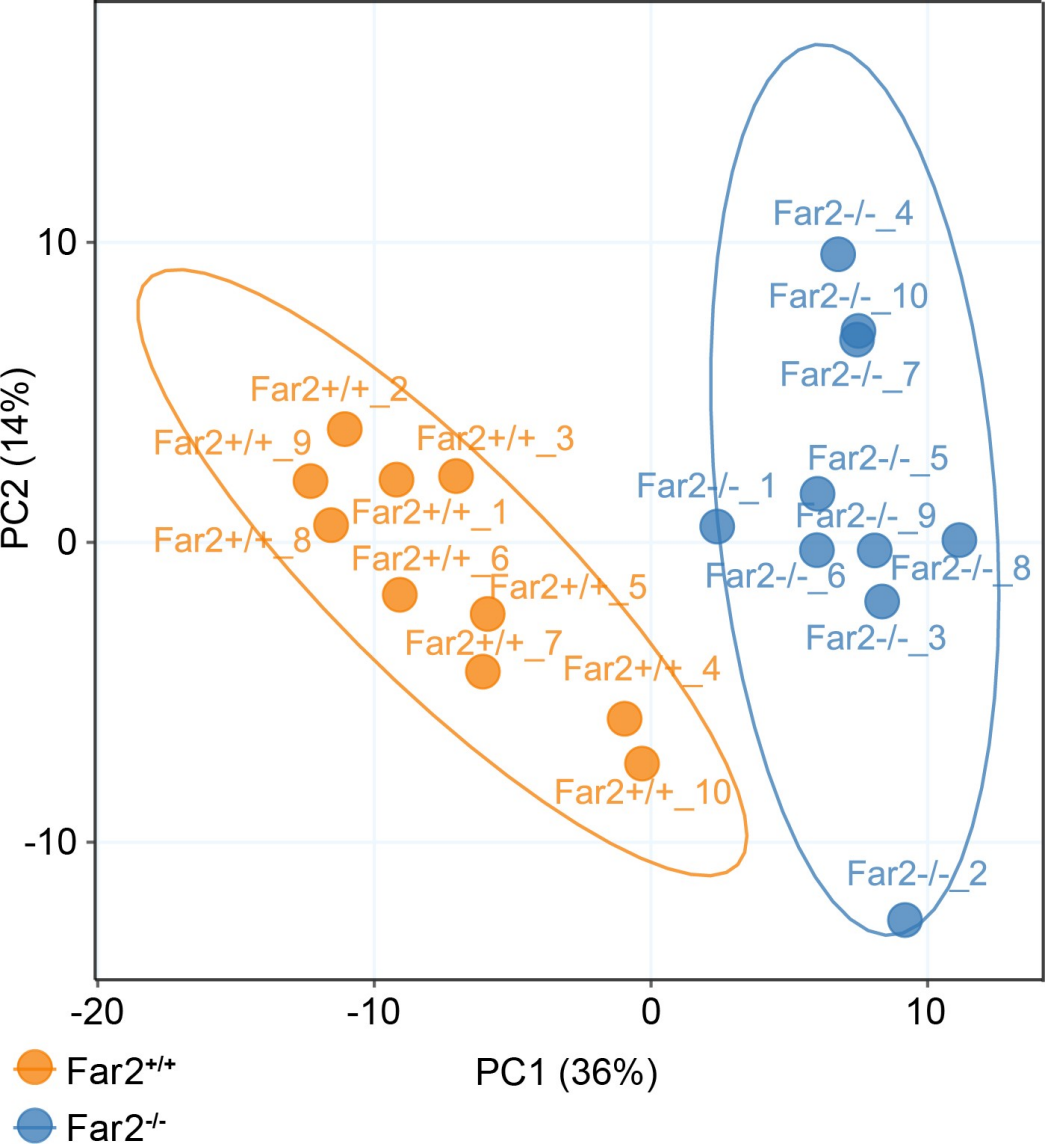
Skin surface lipidomic analyses separate mutant from control mice. Unsupervised multivariate Principal Component Analysis (PCA) suggested distinct differences in metabolomics phenotypes between *Far2*^*-/-*^(N = 10) and *Far2*^+/+_^ mice (N = 10).

**Fig 4:**
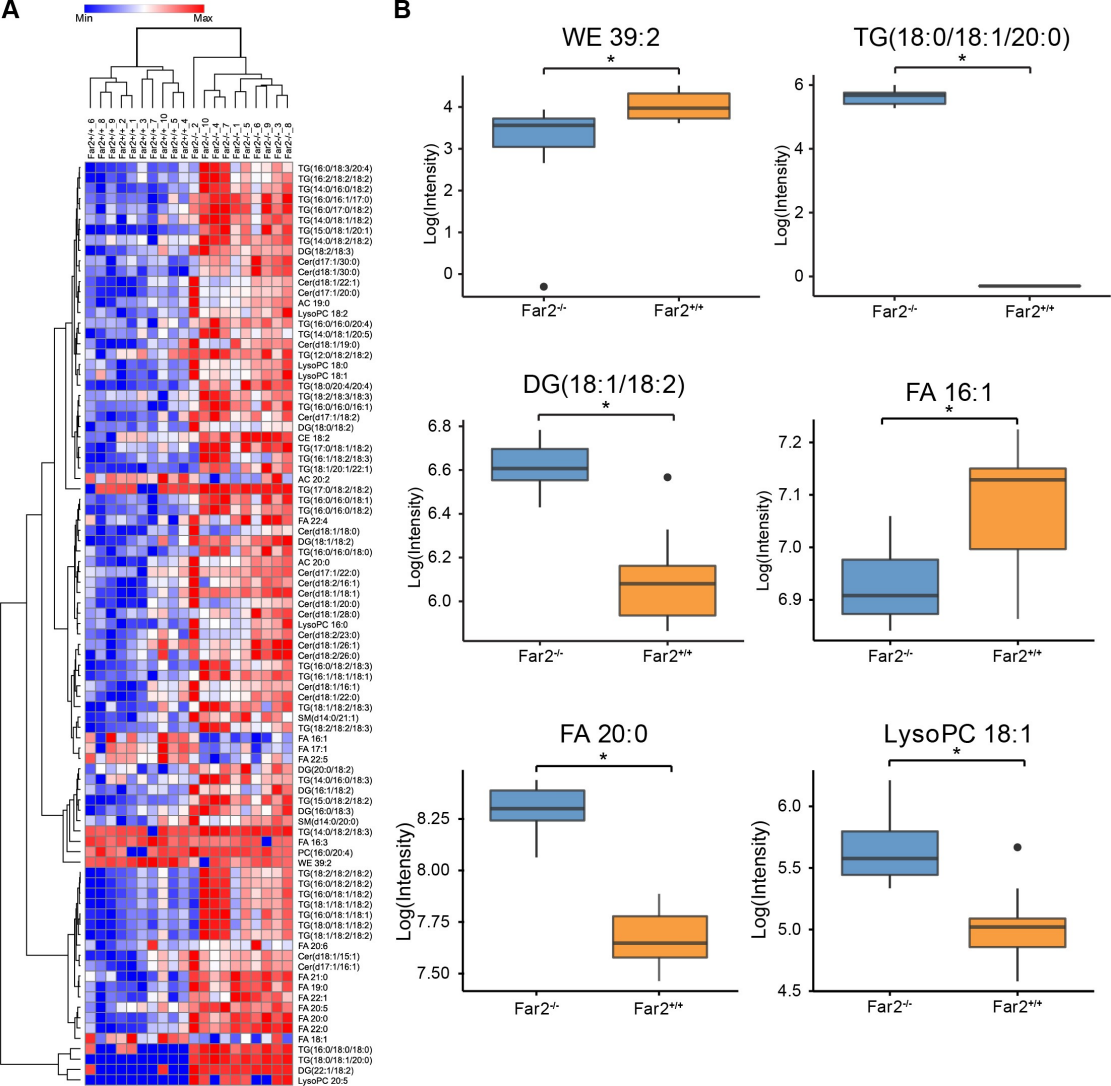
Comparison of skin surface lipids between *Far2*^*-/-*^null and *Far2*^+/+^ wildtype mice. Clustering heat map of 89 significantly different lipids between *Far2*^*-/-*^ null and *Far2*^+/+^ wildtype mice (A). Boxplots of representative lipids illustrates differences between *Far2*^-/-^ and *Far2*^+/+^ mice (B). Statistical significance was determined by Mann-Whitney U test after Benjamini-Hochberg false discovery rate adjustment, *p<0.05; N = 10/group. Abbreviations: WE, wax ester; SFA, saturated fatty acid; SFA, saturated fatty acid; DG, diacylglycerol; TG, triacylglycerol; CE, cholesterol ester; (lyso)PC, (lyso)glycerophosphocholine; Cer, ceramide; SM, sphingomyelin; AC, acylcarnitine.

Comparative lipidomics analyses were conducted to investigate the lipids from the mouse skin surface including neutral lipids, sphingolipids, cholesterol esters, and free fatty acids. An additional liquid chromatography (LC) gradient was adopted in order to extend the coverage to wax esters. Wax esters, composed of long-chain fatty alcohols esterified to fatty acids, have high hydrophobicity; therefore, nonpolar mobile phases were used in an almost isocratic gradient to elute and separate individual wax ester species. The results revealed major changes in skin surface lipids between homozygote mutant *Far2*^*-/-*^ and wildtype (*Far2*^*+/+*^) mice, illustrated by both Principle Component Analysis and Hierarchical Clustering Analysis (Figs 3 and 4A). The skin of *Far2*^*-/-*^ mice contained lower levels of wax monoesters (WEs), unsaturated fatty acids (UFAs), and glycerophosphocholines (PCs) but were higher in saturated fatty acids (SFAs), triacylglycerols (TGs), diacylglycerols (DGs), ceramides (Cers) and (lyso)phosphocholines ((lyso)LPCs) (Fig 4A and 4B). Mann-Whitney U test after BH-FDR adjustment revealed 89 metabolites in skin surface that were significantly different between the *Far2* mutant and wildtype mice (Fig 4A and S1 Table).

## Discussion

Mice that have a severe hypomorphic allelic mutation for fatty acyl CoA reductase 2 develop a relatively mild form of patchy alopecia on the dorsal trunk and diffuse hair thinning on the ventral body surface. This mouse strain was created in an essentially blinded, large scale study to identify the function of genes for which there was little or no information available. While it is not surprising to find abnormal lipid profiles and morphology in sebaceous glands and modified sebaceous glands based on the gene inactivated, there was no advance expectation of such findings [7].

Two fatty acyl-Coenzyme A reductases (FAR1 and FAR2) were described to reduce fatty acids to fatty alcohols. FAR1 preferentially reacted with saturated and unsaturated 16 and 18 carbon fatty acids while FAR2 prefered 16 and 18 carbon saturated fatty acids. Both FAR1 and FAR2 are localized in peroxisomes. *Far1* mRNA was found in many mouse tissues but was highest in mouse preputial glands while *Far2* mRNA was most abundant in Meibomian glands but also in the preputial glands [24, 25]. Peroxisomal fatty acyl-CoA reductase 1 disorder (PFCRD), a primarily neurological disorder in which a subset of patients eventually develop cataracts, is described for *FAR1* mutations in humans [26]; however, no disease has been specifically associated with human *FAR2* null or hypomorphic mutations (https://www.omim.org/entry/616156; accessed 3 Apr 2018). However, in human patients, increased expression of FAR2 is associated with IgA nephropathy, lupus nephritis, and diabetic nephropathy [25].

**Fig 5:**
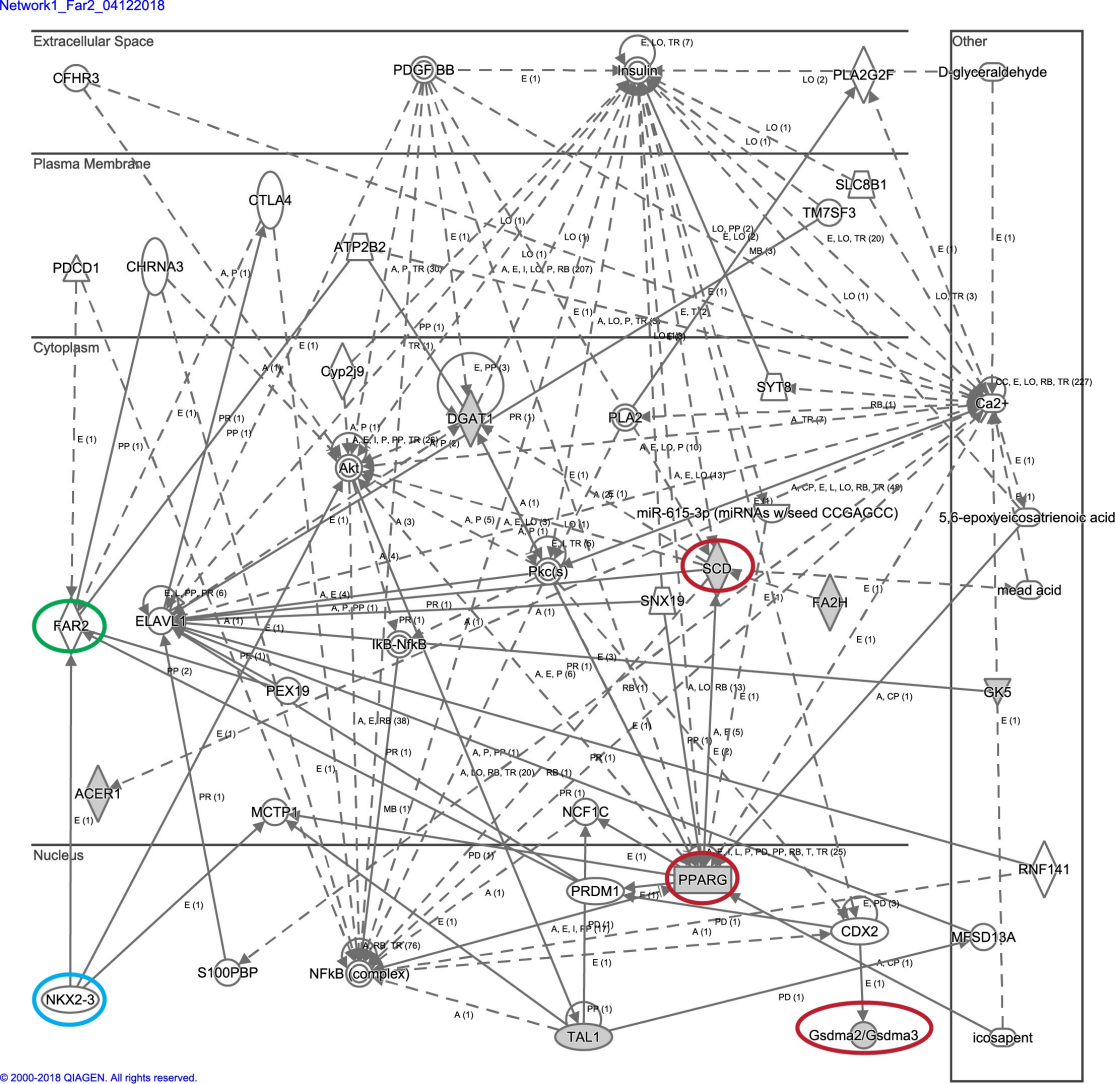
Mouse genes associated with sebaceous gland abnormalities and cicatricial alopecia. SCD1, PPARG, and GSDMA3 mutant mice provide models for scarring alopecia (red circles)[1]. FAR2 is integrated into this pathway (green circle) where it is capable of mediating platelet-activating factor precursors through the NKX3.2 (blue circle) transcription factor [25]. Ingenuity Pathway Analysis (12 April 2018).

In mice lacking functional FAR2 protein, subtle histologic changes in the sebaceous glands as well as significant changes in skin surface lipid composition were observed. More prominent histologic changes as mice aged included follicular dystrophy, rupture, trichogranuloma formation, and eventually scarring to a minimum degree. Changes were similar to those reported in asebia (stearoyl-Coenzyme A desaturase 1, *Scd1*), bareskin (gasdermin A3, *Gsdma3*), and other mutations affecting lipid metabolism resulting in similar morphologic sebaceous gland changes ultimately associated with primary cicatricial alopecias in mice [4, 27–30]. These are but a few mutations in lipid metabolizing enzymes associated with various primary cicatricial alopecias in mice, most of which fall into a complex molecular pathway (Fig 5). Previous work has identified nearly 100 genes that when genetically modified, either mutated or lost, affected the function, development, and/or morphology of the sebaceous gland [31]. Using Ingenuity Pathway Analysis software, many of these genes were fit into an interacting network that may function to influence how the sebaceous gland develops and functions. FAR2 was not automatically linked in this pathway, but further investigation showed that *Far2* mRNA expression is increased through the actions of PRDM1 (BLIMP1; PR domain containing 1, with ZNF domain), a gene that is highly interconnected in the aforementioned sebaceous gland gene set and has been shown to play a role in establishing the size and activity of sebaceous glands [32]. Thus, through the actions of PRDM1, *Far2* can be linked to a much larger sebaceous gland gene network. These findings will begin to expand the complexity of the genetic underpinning of this group of diseases especially since peroxisome proliferator activated receptor gamma (PPARG) is one of the major hubs in the pathway, and also directly influenced by *Prdm1*, and is associated with lichen planopilaris, one of the human primary cicatricial alopecias [5, 33]. Studies on renal glomerular disease propose that FAR2 is involved with *de novo* synthesis of platelet-activating factor (PAF) mediated through NKX3.2 [25].

There are a variety of sebaceous glands in the skin of mammals, not just those associated with hair follicles. The preputial (in males) and clitoral glands (in females) of rodents are specialized sebaceous glands not found in humans. While most sebaceous glands are affected in the asebia mutant mice, the preputial and clitoral glands are not [28]. A spontaneous mutation in the mouse *Hoxd13* gene resulted in mice with normal sebaceous glands in the skin and eyelids but complete absence of preputial and clitoral glands indicating the glands can be under different genetic control for development [34]. Morphologic changes in *Far2* null mice were similar in hair follicle, Meibomian, preputial, and clitoral glands, albeit subtle in the larger glands.

The location of the follicular dystrophy, especially the rupture with trichogranuloma formation, is an important indicator of why follicular scarring was relatively minor. Most of the severe inflammation was located deep within the hypodermal fat layer. This would affect at most the transient amplifying cells of the hair follicle bulb, not the stem cells that are located below the sebaceous glands where the arrector pili muscle attaches. There were scattered granulomas at the level of the stem (bulge) cells and some scarring was present in these areas.

Perilipin 2 (PLIN2) and sterol O-acyltransferase 1 (SOAT1) are two proteins used to identify sebaceous glands in tissue sections while keratin 14 (KRT14) is useful for outlining the gland when investigating its involvement in cancer or for structural abnormalities [12, 35]. Immunohistochemical evaluation of PLIN2 and KRT14 expression revealed regional differences in expression between *Far2*^-/-^ and *Far2*^+/+^ mice while SOAT1 did not. KRT14 was expressed in basal cells lining of the entire pilosebaceous unit but lost from mature disintegrating sebocytes in wildtype mouse hair follicles. By contrast, KRT14 was irregularly expressed along the pilosebaceous epithelium including luminal cells and sebocytes being extruded. PLIN2 was expressed in immature sebocytes at the base of the gland in wildtype mice. By contrast in *Far2*^-/-^ mice PLIN2 was found at the base of the sebaceous glands but also was prominent in sebocytes that were being extruded and as well as in the fully disintegrated cellular extruded material, indicating the immature state of sebocytes on their way out. SOAT expression in the reserve cells of the sebaceous glands was similar in both mutant and control mice. Whether the extruded material within the infundibula was a mixture of lipids (PLIN2) and intermediate filaments (KRT14) was not determined.

The mutant mouse sebocytes had mild discrete electron dense material of various sizes in mitochondria within sebocytes similar to more severe mitochondrial lesions reported in keratinocytes in several scaly skin mutants including chronic proliferative dermatitis (*Sharpin*^*cpdm*^) [23] and flaky skin (*Ttc7*^*fsn*^) [36]. When osmium tetroxide is used in tissue preparation for transmission electron microscopy, labeled lipoproteins appear as electron dense structures. Osmium tetroxide is used to postfix samples and provide electron-dense contrast. It also prevents the extraction of lipids by ethanol and propylene oxide during the dehydration process [37]. As such, these mitochondrial electron dense structures are likely to be an indication of abnormal lipid accumulation. In the chronic proliferative dermatitis mutant mouse (*Sharpin*^*cpdm/cpdm*^), the mitochondrial lesions correlated with the apoptotic pathway being abnormal which was manifest by the large number of keratinocytes undergoing apoptosis [23]. A similar molecular process may be involved in the sebocytes of the *Far2*^-/-^ mice which would account for the abnormal phenotype of the sebocytes. The eosinophilia of sebocytes entering the sebaceous gland duct in H&E stained sections suggested that these sebocytes might be undergoing apoptosis. Cleaved caspase 3 localization in such cells is one way to assess apoptosis. The positive control sample from *Sharpin*^*cpdm/cpdm*^ mutant mice revealed abundant apoptotic keratinocytes [23] (posted in the Mouse Tumor Biology Database, immunohistochemistry, http://tumor.informatics.jax.org/) but none of the sebocytes in the *Far2*^-/-^ skin sections had any positive reactivity suggesting that this is not the mechanism accounting for the sebaceous gland abnormality leading to follicular dystrophy. Alternatively, sebocytes may be undergoing pyroptosis, a process mediated by members of the gasdermin family. Several of the gasdermins work through different caspases, which might explain the negative results looking for expression of cleaved caspase 3 [38, 39]. Mutations in gasdermin A3, that result in sebaceous gland abnormalities and scarring alopecia, *in vitro* result in failure of the repressor domain to inhibit the pore-forming domain resulting pores in membranes causing cells to swell and rupture [39, 40]. Gasdermin A3 or other members of this family may be more primary intermediaries in the pathogenesis of the *Far2*^-/-^ skin disease than is indicated in Fig 5. It is possible that sebum production has not fully matured in mice with sebaceous gland abnormalities leading to scarring alopecias, harmful substances such as oleic acid are released, and at the same time, the part of the pyroptotic pathway that will release lipid inflammatory mediators may still find substantial substrate to work with while the cell is on its way out of the sebaceous gland, releasing pro-inflammatory molecules as it goes. The relative decrease in wax esters on the skin surface might support that.

Non-integument phenotypes affecting lipid metabolism, behavior, immunity, renal function, and the cardiovascular system have been reported for this *Far2*^-/-^ allelic mutation, and this information can be found on the IMPC database website (http://www.mousephenotype.org/data/genes/MGI:2687035#section-associations) [25].

Fatty acyl coA reductase catalyzes the reaction which converts fatty acids to fatty acid alcohols. Lipidomics results reported here demonstrated that after knocking out *Far2* in mice, the level of saturated fatty acids specifically increased, most likely due to unsuccesful conversion to fatty alcohols; whereas, unsaturated fatty acids were lower in *Far2*^*-/-*^ skin compared to that in *Far2*^-/-^ mice, possibly indicating peroxisome dysfunction. Interestingly, such drastically higher level of SFAs included FA long chains ones such as FA 20:0, 22:0, 20:1, and 19:0; yet, changes in FA 16:0 and 18:0 did not reach statistical significance. Cheng and Russell [24] illustrated that human FAR2 prefers FA 16:0 and FA 18:0 as substrates, in contrast to the broad substrate preference of hFAR1. They did not test the substrate specificity toward saturated long chain fatty acids. An enhanced ratio of SFAs to UFAs were also incorporated into acylcarnitines which might account for the mitochondrial densities. Most WEs were significanly lower in the skin of *Far2*^*-/-*^ mice as a result of lower amounts of precursor fatty alcohols.

In other aspects of lipid metabolism, FAR2 also altered the levels of neutral lipids and ceramides, as well as the ratio of LysoPCs/PCs on mouse skin. *Far2*^*-/-*^ mouse skin contained higher TGs, DGs, and Cers compared to wildtype mice. TG (16:0/18:0/18:0) showed a 20-fold increase and TG 18:0/18:1/20:0 were only present in skin of *Far2*^*-/-*^ mice. The absence of FAR2 might subsequently shunt lipid biosynthesis more towards neutral lipids and sphingolipids rather than phospholipids. It is noteworthy that drastically lowered amounts of LysoPCs were observed in *Far2*^*-/-*^ mouse skin, in the opposite trend of PC. FAR might also be associated with lysophosphocholine acyltransferase activity, which enzyme is responsible to convert LysoPCs to PCs.

Earlier studies of mouse models for primary cicatricial alopecia due to sebaceous gland abnormalities utilized thin layer chromatography, a much less precise technology than used in this study, to evaluate skin surface lipids in mutant mice. Asebia mice had significant decreases in wax mono- and diesters and sterol esters but increased free sterols on the skin surface [28, 41]. While there appears to be some overlap, which might help define common molecular pathways, detailed comparisons using the same technology to evaluate multiple mouse models with primary cicatricial alopecias may help to provide novel biomarkers that will enable reliable stratification of the various types.

## Conclusions

*Far2*^-/-^ mice provide further support for a central role for chronic sebadenitis in the development of a number of cicatricial alopecias (in particular those not associated with generalized auto-immune disease-if no auto-antibodies are needed but the released lipid-mix itself becomes more pro-inflammatory). Most human PCAs involve the sebaceous gland, but whether this is the cause or effect remains to be determined.

## Supporting information

S1 Table The significantly changed skin surface lipids between Far2 null mice and wildtype (N = 10). (PDF)

## Abbreviations

AC: acylcarnitine
CE: cholesterol ester
Cer: ceramide
DG: diacylglycerol
*Far2*/FAR2: Fatty acyl CoA reductase 2 (*Gene*/PROTEIN)
FA: free fatty acids
FDR: false discovery rate
KRT14: keratin 14 protein
LC: liquid chromatography
(lyso)PC: (lyso)phosphocholine
PC: glycerophosphocholine
PCA: primary cicatricial alopecia
PLIN2: perilipin 2 protein
SFA: saturated fatty acid
Sharpin^cpdm^: Sharpin gene chronic proliferative dermatitis allelic mutation
SM: sphingomyelin
SOAT1: sterol-O-acyltransferase 1
SQ: squalene
TG: triacylglycerol
TG: triglyceride
Ttc7^fsn^: tetratricopeptide repeat domain 7 gene flaky skin allelic mutation
WE: wax ester
UFA: unsaturated fatty acid
