# Supplementary material for: Sebaceous gland abnormalities in fatty acyl CoA reductase 2 (Far2) null mice result in primary cicatricial alopecia
Source: PLoS One. 2018 Oct 29;13(10):e0205775. doi: 10.1371/journal.pone.0205775 (PMC6205590; doi:10.1371/journal.pone.0205775)
Supplement: S1 Table — (PDF) [file pone.0205775.s001.pdf]

Table S1. The significantly changed skin lipids between *Far2* null mice and wildtype (N=10).

Lipid name	INCHIKEY	Raw p-value	FDR p-value	Fold Change (FC=null/WT)	Log2(FC)
AC 19:0	GWKZIORVZQMWMC-UHFFFAOYNA-O	0.000	0.001	4.2	2.1
AC 20:0	SVJLJQBGUITFLI-UHFFFAOYNA-O	0.000	0.001	6.6	2.7
AC 20:2	OLZWDVKTOGTVLC-UTJQPWESNA-O	0.007	0.016	0.6	-0.7
CE 18:2	NAACPBBQTFFYQB-LJAITQKLSA-N	0.000	0.001	3.0	1.6
Cer(d17:1/16:1)	WPFUBVVMOSGKHP-CBLABHCTNA-N	0.001	0.003	3.3	1.7
Cer(d17:1/18:2)	WVGYQIXEGMIOFM-QCSXBKGRNA-N	0.000	0.001	2.7	1.4
Cer(d17:1/20:0)	CZKJGFHQZINDHS-NHQGMKOONA-N	0.000	0.000	6.1	2.6
Cer(d17:1/22:0)	NHEGYDMEFDPAEY-NWBJSICCNA-N	0.000	0.001	6.2	2.6
Cer(d17:1/30:0)	CCLRBUCIGBPXLN-MZBAEZDSNA-N	0.000	0.001	4.8	2.3
Cer(d18:1/15:1)	OAFLTWKJXZGYSZ-MANHSESENA-N	0.003	0.007	3.5	1.8
Cer(d18:1/16:1)	BCZGPUAIZZZGTF-HSCORPAWNA-N	0.005	0.013	3.4	1.8
Cer(d18:1/18:0)	VODZWWMEJITOND-OWWNRXNENA-N	0.005	0.013	2.2	1.1
Cer(d18:1/18:1)	TZXPILCYVLWMNW-DXXMRVLNNA-N	0.000	0.000	9.2	3.2
Cer(d18:1/19:0)	SNMLDULQFHAZRC-NHQGMKOONA-N	0.001	0.002	2.8	1.5
Cer(d18:1/20:0)	XWBWIAOWSABHFI-QOSDPKFLNA-N	0.009	0.020	2.3	1.2
Cer(d18:1/22:0)	KEPQASGDXIEOIL-LAPDZXRHNA-N	0.012	0.026	3.2	1.7
Cer(d18:1/22:1)	OINPSEGEBLVAKZ-MBXZFYGDNA-N	0.002	0.006	3.7	1.9
Cer(d18:1/26:1)	IBJXLRPUPQTCQS-SJWJHEOENA-N	0.015	0.032	2.6	1.4
Cer(d18:1/28:0)	NEUDBTFZYJPJMG-OHYVAXEENA-N	0.002	0.004	3.2	1.7
Cer(d18:1/30:0)	YTMSJWGKIOCDQT-BOMCYIIUNA-N	0.000	0.000	8.2	3.0
Cer(d18:2/16:1)	CJIOFRYYJRFSRB-UXKXQDCHNA-N	0.001	0.002	7.3	2.9
Cer(d18:2/23:0)	YVMFSKMDQVDKIS-QCVIXQMPNA-N	0.015	0.032	2.5	1.3
Cer(d18:2/26:0)	ZLOXRQZQPDQQBX-NBGJCBSLNA-N	0.001	0.002	4.5	2.2
DG(16:0/18:3)	UHPKYXAUVQOHQL-SVNQLWEDNA-N	0.000	0.001	2.2	1.1
DG(16:1/18:2)	BHGPPCIWDXQOMA-RUGCENDANA-N	0.000	0.001	2.2	1.1
DG(18:0/18:2)	AJMZUFBKADIAKC-MLWYYCKJNA-N	0.000	0.000	1.8	0.9
DG(18:1/18:2)	BLZVZPYMHLXLHG-RQOIEFAZNA-N	0.000	0.001	2.9	1.6
DG(18:2/18:3)	PGXBELQFNRPKBC-DYXXURTGNA-N	0.000	0.001	2.4	1.3
DG(20:0/18:2)	PAZSROGSZMWHFZ-ZARCXLEVNA-N	0.000	0.001	1.8	0.9
DG(22:1/18:2)	ZLQICTBXADUIDP-YZCPFKTINA-N	0.000	0.001	8.4	3.1
FA 16:1	PJHOFUXBXJNUAC-KTKRTIGZSA-N	0.007	0.016	0.7	-0.5
FA 16:3	HECRUYRQDPYOGL-CQXHLCGLSA-N	0.023	0.049	0.2	-2.0
FA 17:1	FJMPAYNWWWCODA-KHPPLWFESA-N	0.019	0.040	0.8	-0.3
FA 18:1	UWHZIFQPPBDJPM-FPLPWBNLSA-N	0.009	0.020	0.7	-0.5
FA 19:0	ISYWECDDZWTKFF-UHFFFAOYSA-N	0.000	0.000	3.2	1.7
FA 20:0	VKOBVWXKNCXXDE-UHFFFAOYSA-N	0.000	0.000	4.1	2.0
FA 20:5	JAZBEHYOTPTENJ-JLNKQSITSA-N	0.000	0.000	1.8	0.9
FA 20:6	PWIBDJLBEKARAO-LJFGOLAKSA-N	0.003	0.007	1.5	0.6
FA 21:0	CKDDRHZIAZRDBW-UHFFFAOYSA-N	0.000	0.000	3.7	1.9
FA 22:0	UKMSUNONTOPOIO-UHFFFAOYSA-N	0.000	0.000	5.8	2.5
FA 22:1	KJDZDTDNIULJBE-VAWYXSNFSA-N	0.000	0.001	1.7	0.8
FA 22:4	IGFLWIHPPADNDL-GFRMADBLSA-N	0.002	0.006	1.8	0.8
FA 22:5	YUFFSWGQGVEMMI-RCHUDCCISA-N	0.001	0.003	0.6	-0.8
LysoPC 16:0	ASWBNKHCZGQVJV-UHFFFAOYNA-N	0.000	0.000	6.6	2.7
LysoPC 18:0	IHNKQIMGVNPMTC-UHFFFAOYNA-N	0.000	0.000	6.6	2.7
LysoPC 18:1	YAMUFBLWGFFICM-SEYXRHQNNA-N	0.000	0.001	4.6	2.2
LysoPC 18:2	SPJFYYJXNPEZDW-UTJQPWESNA-N	0.000	0.000	5.8	2.5
LysoPC 20:5	PDIGSOAOQOXRDU-WMPRHZDHNA-N	0.002	0.006	Indefinite	Indefinite
PC(16:0/20:4)	JAVWFBAAZSHHAD-KKILVFCFNA-N	0.021	0.045	2.2	1.1
SM(d14:0/20:0)	BLHKKGMGTNRNHM-UHFFFAOYNA-N	0.007	0.016	2.9	1.5
SM(d14:0/21:1)	LDJGVBCPOLCJJN-DQRAZIAONA-N	0.000	0.001	2.7	1.4
TG(12:0/18:2/18:2)	MVLRQPPTISTJFW-ZHSZNRNPNA-N	0.002	0.004	3.2	1.7
TG(14:0/16:0/18:2)	HUAFGDPJQVZKDZ-TXMPEXPYNA-N	0.000	0.000	3.1	1.7
TG(14:0/16:0/18:3)	DPOXSBSJPQIZQK-AAPVPQQHNA-N	0.004	0.010	1.7	0.7
TG(14:0/18:1/18:2)	JRCFMQNYNMHGRD-PUWPDXQZNA-N	0.000	0.001	2.5	1.3
TG(14:0/18:1/20:5)	YYZOTXFUVQAQSS-MIMRAKPYNA-N	0.001	0.003	2.1	1.1
TG(14:0/18:2/18:2)	IPZYSMBZEKSFQU-LHMLETAINA-N	0.000	0.000	3.0	1.6
TG(14:0/18:2/18:3)	GLRVHWCFJRDQSX-DTIWWKLMNA-N	0.002	0.006	3.3	1.7
TG(15:0/18:1/20:1)	NEKSMCMHOJEPMZ-NJDMNPJTNA-N	0.000	0.000	3.1	1.6
TG(15:0/18:2/18:2)	DRFFNNCYRJLRRH-HTUQFOBENA-N	0.000	0.000	4.4	2.1
TG(16:0/16:0/16:1)	IXWQOOZEHUNDEZ-FVDSYPCUNA-N	0.000	0.000	2.8	1.5
TG(16:0/16:0/18:0)	DQKMNCLZNGAXNX-UHFFFAOYNA-N	0.000	0.001	4.3	2.1
TG(16:0/16:0/18:1)	ZKEWGTVAIGJNBK-QOCHGBHMNA-N	0.000	0.000	2.3	1.2
TG(16:0/16:0/18:2)	KELXZWJUGNROKO-OCGDUTRDNA-N	0.000	0.000	2.8	1.5
TG(16:0/16:0/20:4)	FTMDHBKIGZUTJX-MFRLHHNRNA-N	0.000	0.000	3.0	1.6
TG(16:0/16:1/17:0)	SORUPHBVMAHYOI-QVTSOHHYNA-N	0.000	0.000	5.1	2.4
TG(16:0/17:0/18:2)	SEJGBQZTRJHHNJ-OCGDUTRDNA-N	0.000	0.000	4.3	2.1
TG(16:0/18:0/18:0)	SDNYRTVJOFMYIW-UHFFFAOYNA-N	0.000	0.001	27.6	4.8
TG(16:0/18:1/18:1)	NJWLXJXDVKOEOP-IKJQKJQYNA-N	0.001	0.003	2.4	1.2
TG(16:0/18:1/18:2)	PFTMZNUFSPJEJR-KDHVXLOFNA-N	0.000	0.001	4.3	2.1
TG(16:0/18:2/18:2)	UAWKISGGTGCUMP-HTUQFOBENA-N	0.001	0.002	4.7	2.2
TG(16:0/18:2/18:3)	DWJFHJOYYJJEBL-IXXGKOKVNA-N	0.001	0.002	4.1	2.0
TG(16:0/18:3/20:4)	VDMGICGWNCJKMY-VQTFCNDTNA-N	0.000	0.001	2.4	1.3
TG(16:1/18:1/18:1)	LUMXEMCKOBNHRP-OQGVBXIJNA-N	0.000	0.000	2.7	1.4
TG(16:1/18:2/18:3)	ZNWMQGQSWLJCNF-YOPYQNEPNA-N	0.003	0.007	3.0	1.6
TG(16:2/18:2/18:2)	GOOWHOLGCYSMJZ-RMLMUKSQNA-N	0.000	0.001	2.3	1.2
TG(17:0/18:1/18:2)	JCUUGUUICZELJG-KDHVXLOFNA-N	0.001	0.003	3.7	1.9
TG(17:0/18:2/18:2)	YWUXDCKWZLDYON-HTUQFOBENA-N	0.000	0.002	5.6	2.5
TG(18:0/18:1/18:2)	YELCKHLYVOPWQI-RVWOKPPWNA-N	0.002	0.004	2.4	1.3
TG(18:0/18:1/20:0)	LXPJIWPRELJYCD-FLFQWRMENA-N	0.000	0.001	Indefinite	Indefinite
TG(18:0/20:4/20:4)	KWIGMCRWEINBIR-PUVHIJDNNA-N	0.000	0.000	8.8	3.1
TG(18:1/18:1/18:2)	XIKRLPVGWJXBJH-QFACYJBGNA-N	0.001	0.003	4.7	2.2
TG(18:1/18:2/18:2)	POVFMDGBZDJLAN-BHFHHPLZNA-N	0.000	0.001	4.2	2.1
TG(18:1/18:2/18:3)	PXFPBVBCKXAEFP-QZOFRBEONA-N	0.000	0.001	10.1	3.3
TG(18:1/20:1/22:1)	QCCDHLIGMOFIAY-AZLQGJPRNA-N	0.000	0.000	5.5	2.5
TG(18:2/18:2/18:2)	YLJZNIRHIYWHQC-CRDRQGJOSA-N	0.000	0.001	4.8	2.3
TG(18:2/18:2/18:3)	AGCXWXCIGWPQGW-FBIPNAFWNA-N	0.000	0.001	3.3	1.7
TG(18:2/18:3/18:3)	XCBDYYPMQUVIAE-FWPKVRRPNA-N	0.000	0.001	2.7	1.4
WE 39:2		0.007	0.016	0.3	-1.8
